# Hepatic stellate cell reprogramming via exosome-mediated CRISPR/dCas9-VP64 delivery

**DOI:** 10.1080/10717544.2020.1850917

**Published:** 2020-12-18

**Authors:** Nianan Luo, Jiangbin Li, Yafeng Chen, Yan Xu, Yu Wei, Jianguo Lu, Rui Dong

**Affiliations:** a Department of General Surgery, Tangdu Hospital, Fourth Military Medical University, Xi’an, China; b Department of General Surgery, 943 Hospital of PLA, Wuwei, China; c Department of Breast Surgery, Enshi Central Hospital, Enshi, China

**Keywords:** Exosomes, CRISPR/dCas9-VP64 system, specific delivery, HSCs, hepatocyte nuclear factor 4α

## Abstract

Hepatic stellate cells (HSCs) play a crucial role in the progression of liver fibrosis, which can be considered as the specific therapeutic target of anti-fibrotic treatment. Targeted induction of HSCs to hepatocytes via delivery of clustered regularly interspaced short palindromic repeats (CRISPR)/CRISPR-associated protein 9 (dCas9) system holds promise for hepatic fibrosis treatment. Our study here revealed that CRISPR/dCas9-VP64 system encapsulated in AML12 cell-derived exosomes could efficiently and successfully be delivered into the HSCs. In turn, the CRISPR/dCas9-VP64 system loaded in the exosomes can be efficiently released into the HSCs. As a proof-of-concept study, gRNA against hepatocyte nuclear factor 4α (HNF4α) together with the delivery of CRISPR/dCas9-VP64 system induced the HSCs to hepatocyte-like phenotype. In conclusion, our study here revealed that CRISPR/dCas9-VP64 system encapsulated in AML12 cell-derived exosomes could be functional in HSCs, emerging as a gene therapy strategy for hepatic fibrosis.

## 1. Introduction

Liver fibrosis is the pathological reaction of various chronic pathogenic factors acting on the liver and causing tissue injury and repair, and it is the necessary stage of the development of many liver diseases to cirrhosis (Forbes & Newsome, 2016; Kyritsi et al., 2020). Studies have shown that hepatic fibrosis is a reversible process (Deng et al., 2018; Zhang et al., 2020). In recent years, with the development of molecular biology and the elucidated mechanism of liver fibrosis, gene therapy is expected to be an effective anti-liver fibrosis method. Gene therapy for hepatic fibrosis mainly plays a role in preventing the development of fibrosis, stimulating the division of liver cells and the reconstruction of liver tissue structure (Iredale, 2004; Greuter & Shah, 2019; Choi et al., 2020).

All the cells in the liver, including portal area fibroblasts, hepatocytes, Kupffer cells, hepatic sinus endothelial cells, biliary epithelial cells, and HSCs, have found involved in the process of fibrosis (Aizarani et al., 2019). Among them, HSCs are intensively studied. HSC is a non-parenchymal cell of the liver, accounting for about 15% of the total number of normal hepatic cells. It is located in the disse space between hepatic sinus endothelial cells and hepatocytes, and the cytoplasm is rich in vitamin A and lipid droplets (Tsuchida & Friedman, 2017). When the liver is damaged, HSC turns into myofibroblast, and releases cytokines through autocrine and paracrine to promote the continuous proliferation and activation of itself, and continuously secrete collagen fibers to promote the formation of liver fibrosis (Tao et al., 2019). HSC has been proved to be the main source of extracellular matrix (ECM), and the activation and transformation of HSC into myofibroblast (MFB) is of the key cellular event in hepatic fibrosis. Studies have confirmed that the reversal of liver fibrosis is accompanied by a large number of HSC apoptosis and a significant reduction in the number of cells, and all fibrogenic factors take HSC as the final target cell (DeRossi et al., 2019). HSC targeting by nanocarriers is a promising approach to treat hepatic fibrosis.

Exosome is a membrane-structured nanovesicle secreted by cells to extracellular matrix, which is considered as a ‘bridge’ between cells (van de Vlekkert et al., 2019). Exosomes are 30–150 nm in diameter and can carry a series of complex bioactive substances, which are important mediators for signal transduction between cells and exert their biological effects through complex mechanisms of action (O’Brien et al., 2020). Exosomes, as carriers of intercellular signal transduction and substance transfer, can specifically bind to target cells, deliver their cargos to target cells, and regulate various physiological functions of target cells, such as protein expression, cell proliferation and differentiation, and immune response (Daassi et al., 2020). In recent years, with the increasing research on exosomes, their role in regulating liver fibrosis has also been gradually concerned, and as a carrier of intercellular signal transmission to affect the fibrosis process (Chen et al., 2018; Zhang et al., 2020). Many kinds of cells in liver can secrete exosomes or be target cells of exosomes. Exosomes secreted by different cells have different functions. For example, exosomes from hepatocytes can regulate liver cell proliferation and liver regeneration (Wu et al., 2018), and exosomes from HSCs are involved in the formation of liver fibrosis. As a natural carrier of targeted therapy, exosomes can specifically deliver therapeutic agents and targeted peptides to HSCs, so as to achieve targeted therapy of hepatic fibrosis (Babuta et al., 2019; Reshke et al., 2020).

Gene editing is a technique for fixed-point modification of DNA nucleotide sequences, which can precisely cut off targeted DNA fragments and insert new gene fragments (Kaufmann et al., 2013). It is an important technique in genetic engineering. The emergence of CRISPR/dCas9 technology marks the entry of gene editing technology into a new field, providing great help for the research of gene function and the treatment of diseases (Chang et al., 2018). CRISPR/dCas9 system is a technology for specific DNA modification of targeted genes by RNA-guided Cas nucleases (Himeda et al., 2016). This new high-specificity and efficient gene editing technology can treat a variety of diseases through DNA cutting technology, which opens a new direction for gene therapy. The CRISPR/dCas9-based gene-editing technology has shown great promise in a range of applications of gene therapy, such as blood diseases, tumors and other genetic diseases (Rousset et al., 2018). The technique has been used to modify the genomes of human cells, zebrafish, mice and bacteria. With the advantages of low cost, convenient operation and high efficiency (Cress et al., 2016), CRISPR/dCas9 technology has quickly become popular in laboratories all over the world and become a powerful helper for biological research (Xiao et al., 2019). The biggest breakthrough of this technology is that it can edit not only a single gene, but also many genes at the same time, which provides an effective method for whole-genome screening. CRISPR/dCas9 technology can knock out, edit, inhibit and activate genes, and CRISPR/dCas9-VP64 system has an efficient gene transcriptional activation function in this study (Konermann et al., 2015). The rapid development of CRISPR/dCas9 technology has brought light to gene therapy for liver fibrosis.

In this study, we constructed the AML12 cell-derived exosome delivery system loading the CRISPR/dCas9-VP64 system, and the exosomes successfully delivered the CRISPR/dCas9-VP64 to HSCs. As a proof-of-concept study, HNF4α gene was efficiently posttranscriptional activated by the CRISPR/dCas9-VP64 mediated activation system in HSCs, while HNF4α is a transcriptional regulatory factor of hepatocytes differentiation and HNF4α could significantly attenuate hepatic fibrosis (Yue et al., 2010). This study lays a foundation for the HSCs in situ reprogramming and provides new ideas and strategies for targeted gene therapy of liver fibrosis.

## 2. Materials and methods

### 2.1. Cell culture

Mouse liver AML12 cells, HEK293T cells, RAW264.7 cells and mouse HSCs were cultured in high-glucose Dulbecco’s modified Eagle medium (DMEM) containing 10% fetal bovine serum (FBS) and 1% penicillin/streptomycin (Logan, UT). RAW264.7 cells were maintained in 1640 medium (Logan, UT) with 10% FBS and 1% antibiotics. Cells were changed with fresh medium every other day and grown at 37 °C in a 5% CO_2_ atm.

### 2.2. Animal housing

C57BL/6 mice (6–8 weeks old) were obtained from the Lab Animal Center of Fourth Military Medical University. All mice were housed under specific-pathogen-free conditions. The experimental procedures were performed in accordance with the guidelines approved by the Institutional Animal Experiment Administration Committee of the Fourth Military Medical University. All mice were euthanized for liver tissues harvest.

### 2.3. Plasmid construction

sgRNAs targeting HNF4α gene or the control were designed using the online CRISPR Design Tool (http://tools.genome-engineering.org). The synthesized paired oligos were diluted in sterile water and annealed in a thermal cycler. The annealed oligos were then cloned into the lenti sgRNA backbone after BsmBI digestion. The referred primer sequences for plasmid construction are listed in Supplementary Table S1.

### 2.4. Lentivirus packaging and infection

The lentivirus expressing dCas9 or sgRNA were transfected into HEK293FT cells together with another two plasmids psPAX2 and pMD2G at a 4:3:1 ratio. The three plasmids dissolved in FBS-free DMEM medium were mixed with Lipofectamine 2000 (Invitrogen, Carlsbad, CA) and incubated at room temperature for 20 min. Then, HEK293FT cells at 70–80% confluence received the transfection, and the medium was changed with fresh 10% FBS-containing medium 6 h later. Lentivirus particles were collected from the medium supernatant filtered through a 0.45 μm filter (Millipore, Shanghai, China) 48 h post transfection and stored at −80 °C before use.

### 2.5. Exosome isolation and characterization

AML12 cells were cultured with full medium at 80–90% confluency replaced with fresh medium without FBS. After 48 h culture, the cell medium was harvested and centrifuged at 500 *g* for 10 min to remove cells and then at 10,000 g for 20 min to eliminate the residual cellular debris. The resulting supernatant was regularly filtered through 0.22 μm filters. Exosomes were isolated from the cell medium using Exoquick-TC™ kit (System Biosciences, Johnstown, PA). The mixture was then incubated at 4 °C overnight and centrifuged at 12,000 *g* for 1 h. Exosome pellets were resuspended in PBS or DMEM and stored at −80 °C.

For transmission electron microscopy analysis of the exosomes, isolated exosomes were added on a copper grid and were stained with phosphotungstic acid 10 min later. The dried grids were examined using the transmission electron microscope (JEM-2000EX TEM, JEOL Ltd, Tokyo, Japan).

For size distribution analysis of the exosomes, isolated exosomes were diluted to 500 ng/ml and subjected for size distribution analysis.

### 2.6. Western blotting

Total protein from cells, tissues or exosomes was extracted using RIPA Lysis Buffer (Beyotime Biotechnology, Beijing, China) at 4 °C for 30 min. Protein concentration was determined by Pierce BCA Protein Assay Kit (Thermo, Waltham, MA). Protein samples were separated by 10% sodium dodecyl sulfate polyacrylamide gel electrophoresis and electrotransferred onto the nitrocellulose filter membrane (NC membrane). The NC membrane was incubated with primary antibodies against GM130 (1:1000; Abcam, Shanghai, China); CD63 (1:1000; Abcam, Shanghai, China), TSG101 (1:1000; Abcam, Shanghai, China), CRBP1 (1:1000; Santa Cruz, Shanghai, China), HNF4α (1:1000; Abcam, Shanghai, China), Cas9 (1:1000; Cell Signaling Technology, Boston, MA), E-Cadherin (1:1000; Abcam, Shanghai, China), α-SMA (1:1000; Abcam, Shanghai, China), collagen I (1:1000; Abcam, Shanghai, China), and GAPDH (1:1500; Cell Signaling Technology, Boston, MA) at 4 °C for 12 h, followed by incubation with the secondary antibody in Tris-buffered saline at room temperature for 1 h. The images were developed by chemiluminescence (GE Healthcare, Buckinghamshire, UK) in a dark room.

### 2.7. Quantitative real-time polymerase chain reaction

Total RNA was isolated using the Trizol (Roche, Basel, Switzerland) before being subjected to reverse transcription using PrimeScript RT Reagent kit (Takara, Tokyo, Japan). Quantitative real-time PCR analysis (qPCR) was conducted with SYBR Premix Ex Taq II (Takara, Tokyo, Japan) using FastStart Essential DNA Green Master (Indianapolis, IN). The mRNA expression was normalized to GAPDH. The referred primer sequences are listed in Supplementary Table S1.

### 2.8. DiI/DiR labeling of exosomes

The red-fluorescent lipophilic dye DiI/DiR (Beyotime Biotechnology, Beijing, China) was used to label the exosomes for in vivo and in vitro tracking, which is incorporated in the outer membrane of exosomes. Briefly, 500 μL of the exosomes (with the protein concentration at 80 μg/ml) is incubated with 2 μl of 10 μM stock solution of DiI/DiR. The unbound free dye was removed by centrifuging the exosomes. For in vivo tracking exosomes, the DiR labeled exosomes (100 μg per mouse, at 100 μL in volume) were injected via tail vein. For in vitro tracking exosomes by fluorescence microscopy, DiI labeled exosomes were used. The nuclei were counterastained with Hoechst.

### 2.9. Immunofluorescence staining

For the immunofluorescence staining of the cells, the cells were fixed by 4% paraformaldehyde for 15 min. Then, the cells were incubated with CRBP1 (1:50; Santa Cruz, Shanghai, China), HNF4α (1:500; Abcam, Shanghai, China), which shows the morphology of HSCs or AML12 cells for 16 h. Then stained with Hoechst (1:1000; Invitrogen, Carlsbad, CA) for counterstaining of the cell nuclei for 15 min. Images were taken with a Nikon A1 Spectral Confocal Microscope (Nikon, Tokyo, Japan).

For the immunofluorescence staining of tissues, the mice were sacrificed at the indicated time, liver tissues from the mice were harvested for sliced sections. For slice section staining, the specimen of liver tissues was immediately harvested and put in 4% paraformaldehyde for 24 h. Next, the tissues were embedded in optimal cutting temperature compound (OCT, Tissue-Tek, Torrance, CA). After sectioning, the tissues on the slides were repaired by antigen retrieval and then stained with CRBP1 (1:50; Santa Cruz, Shanghai, China), HNF4α (1:500; Abcam, Shanghai, China), which shows the morphology of HSCs or AML12 cells for 16 h. Then stained with Hoechst (1:1000; Invitrogen, Carlsbad, CA) for counterstaining of the cell nuclei for 15 min. The whole process was conducted in dark. Images were taken with a Nikon A1 Spectral Confocal Microscope (Nikon, Tokyo, Japan).

### 2.10. Statistical analysis

All the data are expressed as mean ± SEM. Student’s *t* test was used for two group comparison, and one-way ANOVA was used for multiple comparisons by Tukey’s post hoctest (Graphpad Prism 6.0, GraphPad Software, La Jolla, CA). *p* Values <.05 was considered statistically significant.

## 3. Results and discussion

### 3.1. Results

#### 3.1.1. Isolation and characterization of AML12 cell-derived exosomes

**Figure 1.**
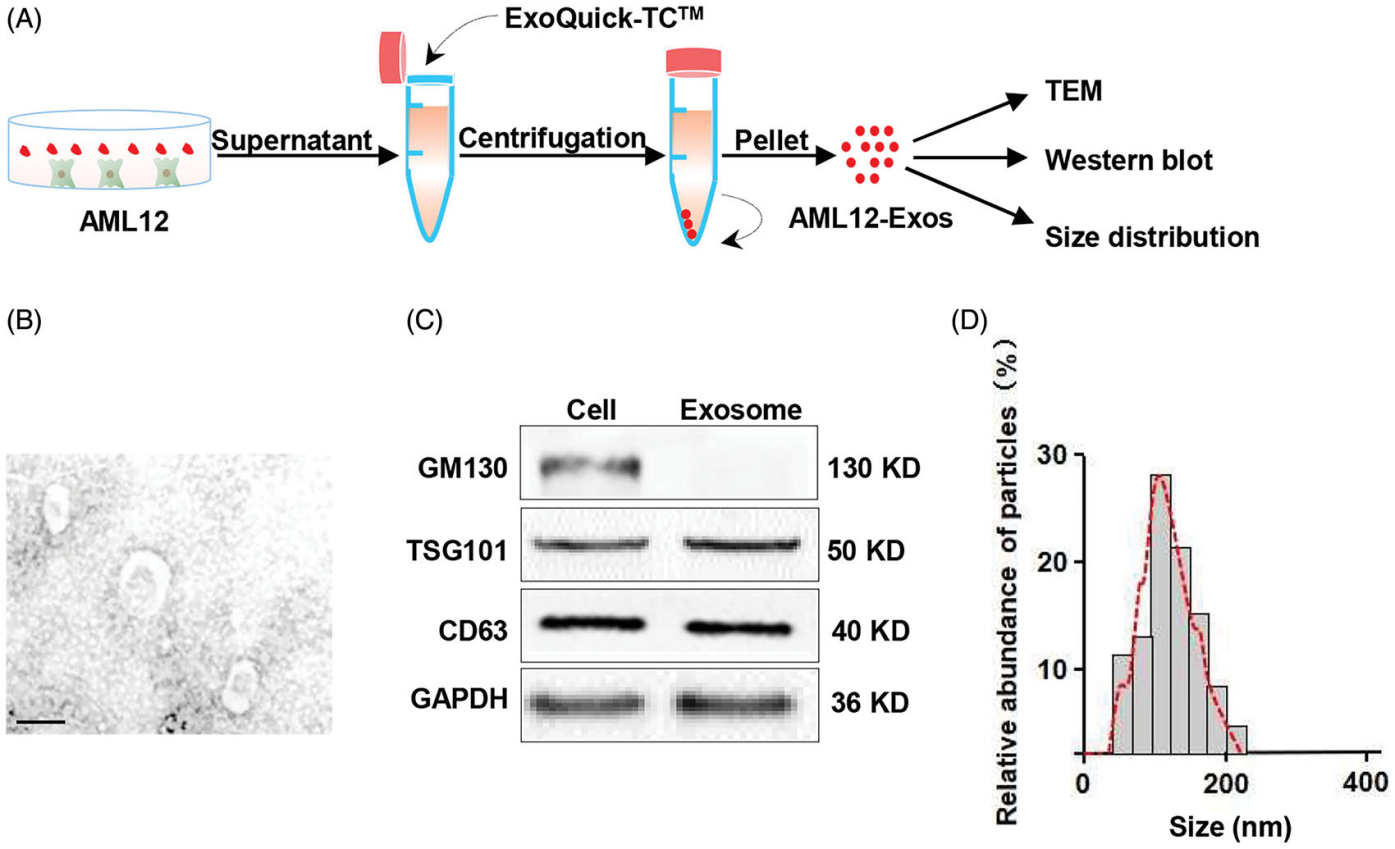
Isolation and identification of AML12 cell-derived exosomes. (A) Schematic representation of the exosomes isolation procedure; (B) representative TEM (transmission electron microscope) image of the isolated exosomes (scale bar = 100 nm); (C) western blot analysis of the expression of exosomal markers GM130, TSG101 and CD63 in the isolated exosomes and the parental cells. GAPDH served as the loading control; (D) size distribution of the isolated exosomes.

Exosomes have been recognized as a promising drug carrier for targeted delivery, we thus tried to encapsulate the CRISPR/dCas9-VP64 system into AML12-Exos and deliver the CRISPR/dCas9-VP64 system to HCSs. We here chose AML12 cells as the source of exosomes for the following reasons: It has been reported that exosome nanovesicles from hepatocytes promote hepatocyte proliferation in vitro and liver regeneration in vivo, and as the endogenous exosomes, AML12-Exos would be safer and more effective. AML12 cells were cultured in the serum free medium and the secreted exosomes were isolated from the culture supernatants (Figure 1(A)). Transmission electron microscope analysis of the exosomes confirmed the saucer-shaped morphology with visible lipid layer (Figure 1(B)). The exosomal markers GM130, TSG101 and CD63 were detected in the exosomes (Figure 1(C)), confirming the identity of the isolated exosomes. Size distribution analysis further verified that the size ranged from 50 to 200 nm in diameter (Figure 1(D)).

#### 3.1.2. Activation of HNF4α gene via CRISPR/dCas9-VP64 system

**Figure 2.**
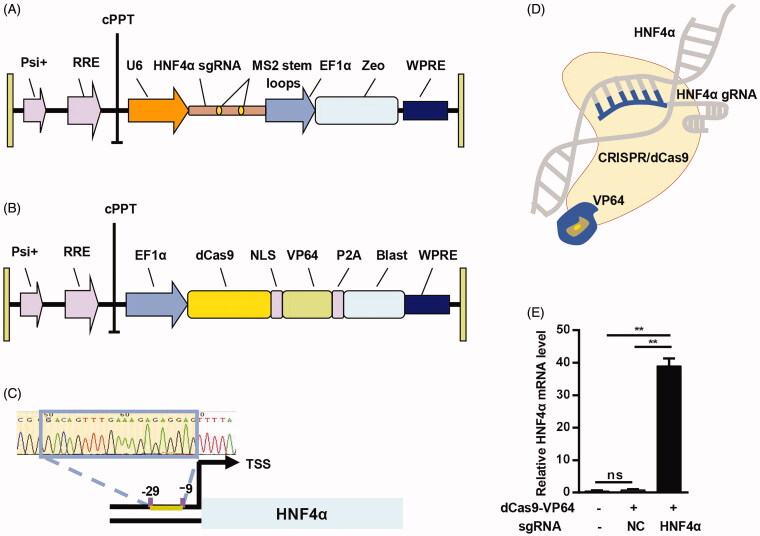
Activation of HNF4α gene by CRISPR/dCas9-VP64. (A) Schematic representation of the lentivirus expressing HNF4α sgRNA; (B) schematic representative of the lentivirus expressing dCas9; (C) sequencing result of the HNF4α sgRNA vector construction; (D) illustration of the CRISPR/dCas9-VP64 mediated activation of HNF4α; (E) expression of HNF4α mRNA in HSCs treated as indicated was analyzed by qPCR. Negative control served as NC. Data are expressed as mean ± SEM of three different experiments. **p < .01.

As an emerging tool for gene editing, CRISPR/dCas9 system possesses some advantages compared to the conventional nuclease tools. Here we used the CRISPR/dCas9-VP64 system to activate gene expression, which is considered to be robust, specific, and could facilitate genomescale gain-of-function screening (Konermann et al., 2015). The CRISPR gene activation system consisted of two vectors, Lenti-sgRNA-zeo and Lenti-dCas9-VP64-blastine (Figure 2(A,B)). In the system, the sgRNA recognizes the complementary sequence of targeting gene, guiding the dCas9-VP64 to the target gene. To activate HNF4α, whose upregulation might present as an ideal option for the treatment of hepatic fibrosis. HNF4α sgRNA construction was confirmed by DNA sequencing (Figure 2(C,D)). As expected, the system successfully activated HNF4α gene expression in HSCs, as revealed by qPCR analysis (Figure 2(E)). Therefore, the CRISPR/dCas9-VP64 system was successfully constructed and can activate the target gene expression.

#### 3.1.3. Aml12-Exos efficiently encapsulate CRISPR/dCas9-VP64 system

**Figure 3.**
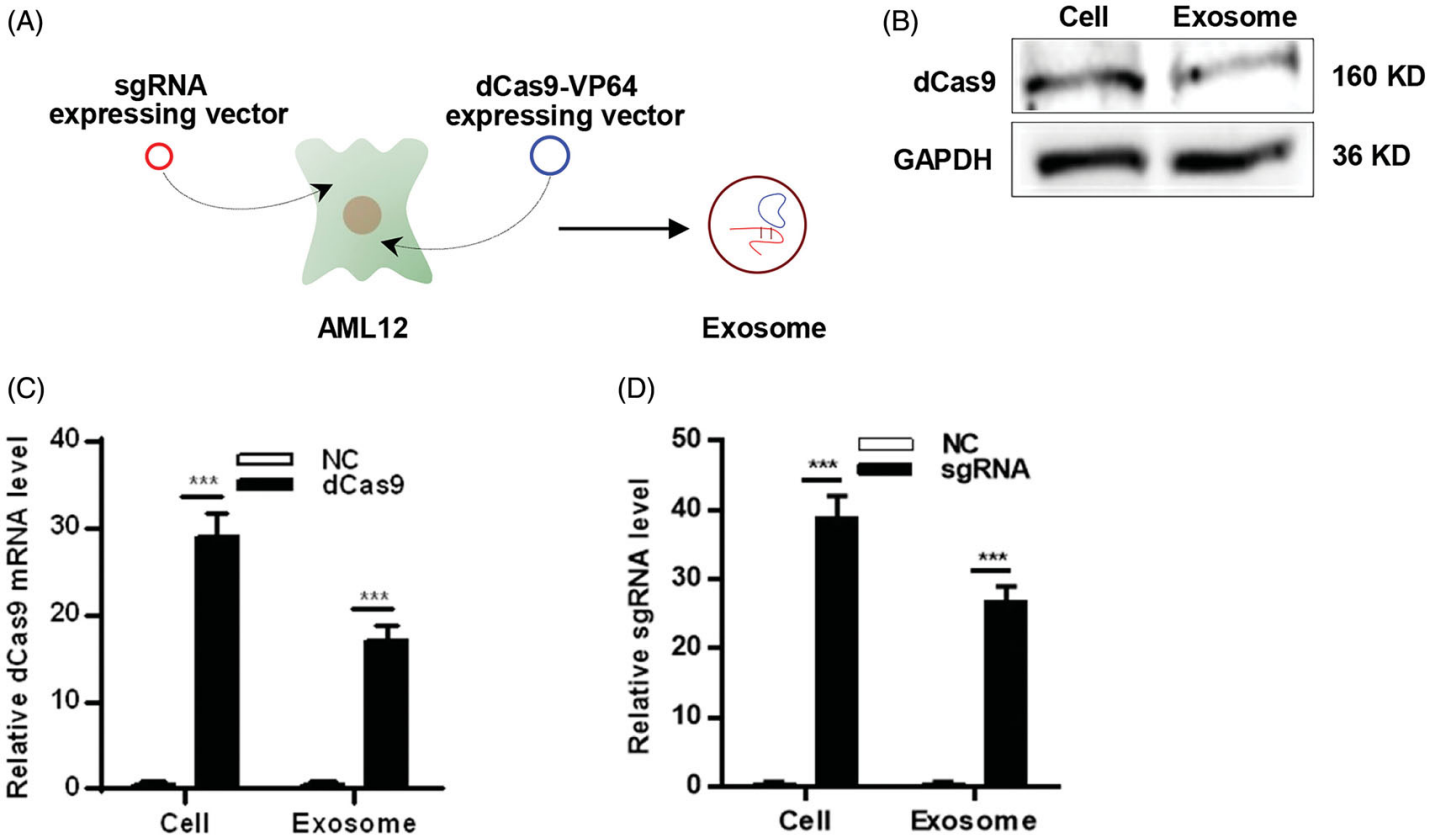
AML12-Exos efficiently encapsulate CRISPR/dCas9-VP64 system. (A) Schematic representation of the exosomes encapsulation procedure; (B) western blot analysis of the expression of protein dCas9 in the isolated exosomes and the parental cells. GAPDH served as the loading control; (C) expression of dCas9 mRNA in the isolated exosomes and the parental cells was analyzed by qPCR; (D) expression of sgRNA in the isolated exosomes and the parental cells was analyzed by qPCR. Negative control served as NC. Data are expressed as mean ± SEM of three different experiments. ***p < .005.

Exosomes have been used as drug delivery vehicles and transferred various cargos. It was reported that plasmid DNA could be loaded into exosomes via transfection of donor cells (Koh et al., 2017). In the following experiments, we attempted to load the CRISPR/dCas9-VP64 plasmid DNA into exosomes, so we isolated exosomes derived from sgRNA and dCas9 expressing AML12 cells (Figure 3(A)). Western blot analysis revealed that dCas9 protein could be examined in the exosomes (Figure 3(B)). Moreover, qPCR analysis of dCas9 mRNA and sgRNA level in the AML12-Exos (Figure 3(C,D)), suggesting that the CRISPR/dCas9-VP64 plasmid DNA was mostly loaded in the exosomes. These data indicate that AML12-Exos efficiently encapsulate the CRISPR/dCas9-VP64 system.

#### 3.1.4. Validation and characterization of AML12 cells and HSCs

**Figure 4.**
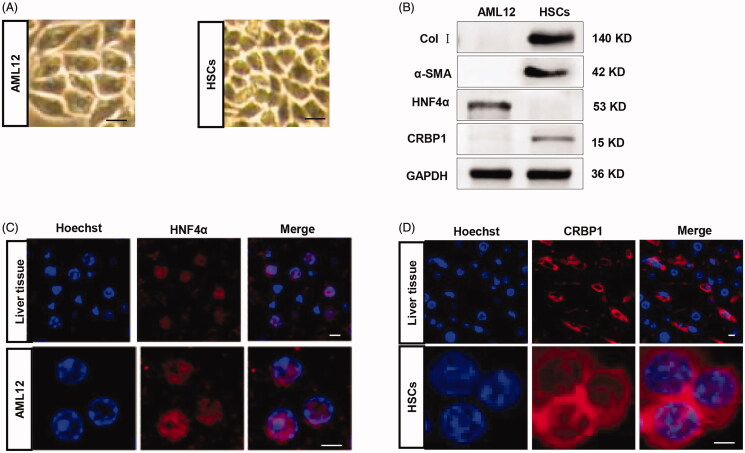
Validation and characterization of AML12 cells and HSCs. (A) Microscope image of the AML12 and HSC cells morphology; (B) western blot analysis of the expression of HNF4α/CRBP1 and α-SMA/collagen I in AML12 cells and HSCs. GAPDH served as the loading control. (C) immunofluorescence microscope image of the expression of HNF4α in the liver tissues and AML12 cells (scale bar = 5 μm). Nuclei were counterstained with Hoechst; (D) immunofluorescence microscope image of the expression of CRBP1 in the liver tissues and HSCs (scale bar = 2.5 μm). Nuclei were counterstained with Hoechst.

In this study, we focused on the AML12 cells and HSCs, the reasons have already been clarified. In order to support later experiments, we tried to validate the AML12 cells and HSCs, and detect the markers of the two cell lines. For AML12 cells, microscope analysis of the morphology revealed that this cell line was spindle-shaped (Figure 4(A)). In the same way, we identified and validated the HSCs, the observations indicated that the morphology of HSCs was star-shaped (Figure 4(A)). Western blot analysis of hepatocyte marker HNF4α and HSC marker CRBP1, alpha-smooth muscle actin (α-SMA) and collagen I, further confirmed the cell identities (Figure 4(B)). Notably, there were robust α-SMA and collagen I expression in HSCs, suggesting that the HSCs used were at least partially activated. Immunofluorescence staining analysis of the AML12 cells and liver tissues found that the HNF4α protein was in the nucleus, further validating the trait of the cells (Figure 4(C)), and the CRBP1 protein was in the cytoplasm of the HSCs (Figure 4(D)). 

#### 3.1.5. Aml12-Exos effectively deliver CRISPR/dCas9-VP64 system into HSCs in vitro and in vivo

**Figure 5.**
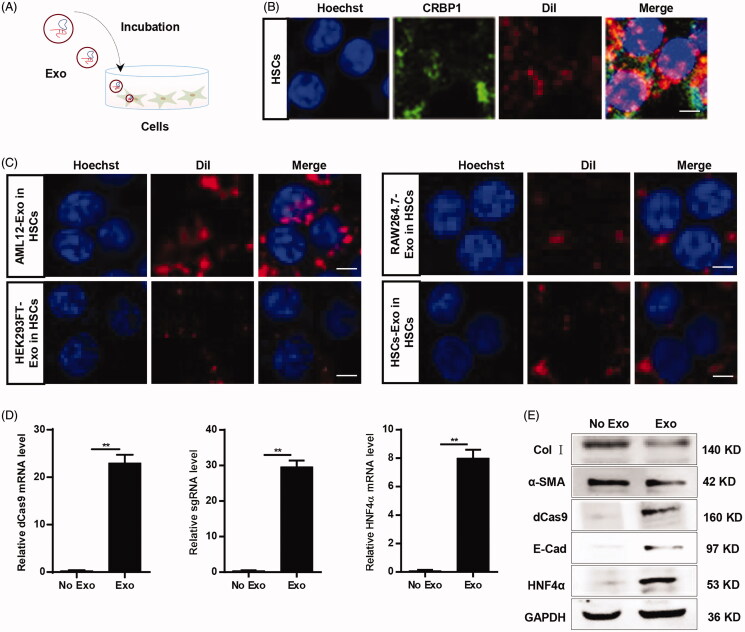
AML12-Exos effectively deliver CRISPR/dCas9-VP64 system into HSCs in vitro. (A) Schematic representation of the exosomes endocytosis procedure by the recipient cells; (B) immunofluorescence microscope image of the colocation of DiI-labeled exosomes and CRBP1 in HSCs (scale bar = 5 μm). Nuclei were counterstained with Hoechst; (C) immunofluorescence microscope image of the different cells-derived DiI-labeled exosomes endocytosis efficiency by the HSCs (scale bar = 5 μm). Nuclei were counterstained with Hoechst; (D) expression of dCas9 mRNA, sgRNA, HNF4α mRNA in the HSCs treated as indicated was analyzed by qPCR. Data are expressed as mean ± SEM of three different experiments. **p < .01; (E) western blot analysis of the expression of dCas9/HNF4α/E-Cadherin and α-SMA/collagen I in the HSCs treated as indicated. GAPDH served as the loading control.

In light of the above data, we next explored whether the CRISPR/dCas9-VP64 system in the exosomes could be endocytosed by the recipient cells in vitro. HSCs were incubated with the exosomes isolated from infected AML12 cells (Figure 5(A)). Immunofluorescence microscope analysis indicated that DiI-labeled exosomes and CRBP1 colocated in the cytoplasm of HSCs (Figure 5(B)), confirming that AML12-Exos at least could enter the recipient cells. Compared with other recipient cells, including AML12 cells, HEK293FT cells, RAW264.7 cells (Figure 5(C), Supplementary Figure S2), HSCs had higher uptake efficiency. qPCR analysis of dCas9 mRNA, sgRNA and HNF4α mRNA level in the HSCs indicated that the CRISPR/dCas9-VP64 system could be effectively delivered into HSCs and work in the recipient cells (Figure 5(D)). Western blot analysis revealed that HNF4α, E-Cadherin and dCas9 were detected, while α-SMA/collagen I production were reduced in HSCs treated with the exosomes (Figure 5(E)). Notably, the observed α-SMA/collagen I expression in the HSC suggested that the engineered exosomes just partially induce the transdifferentiation of HSCs to hepatocytes.

**Figure 6.**
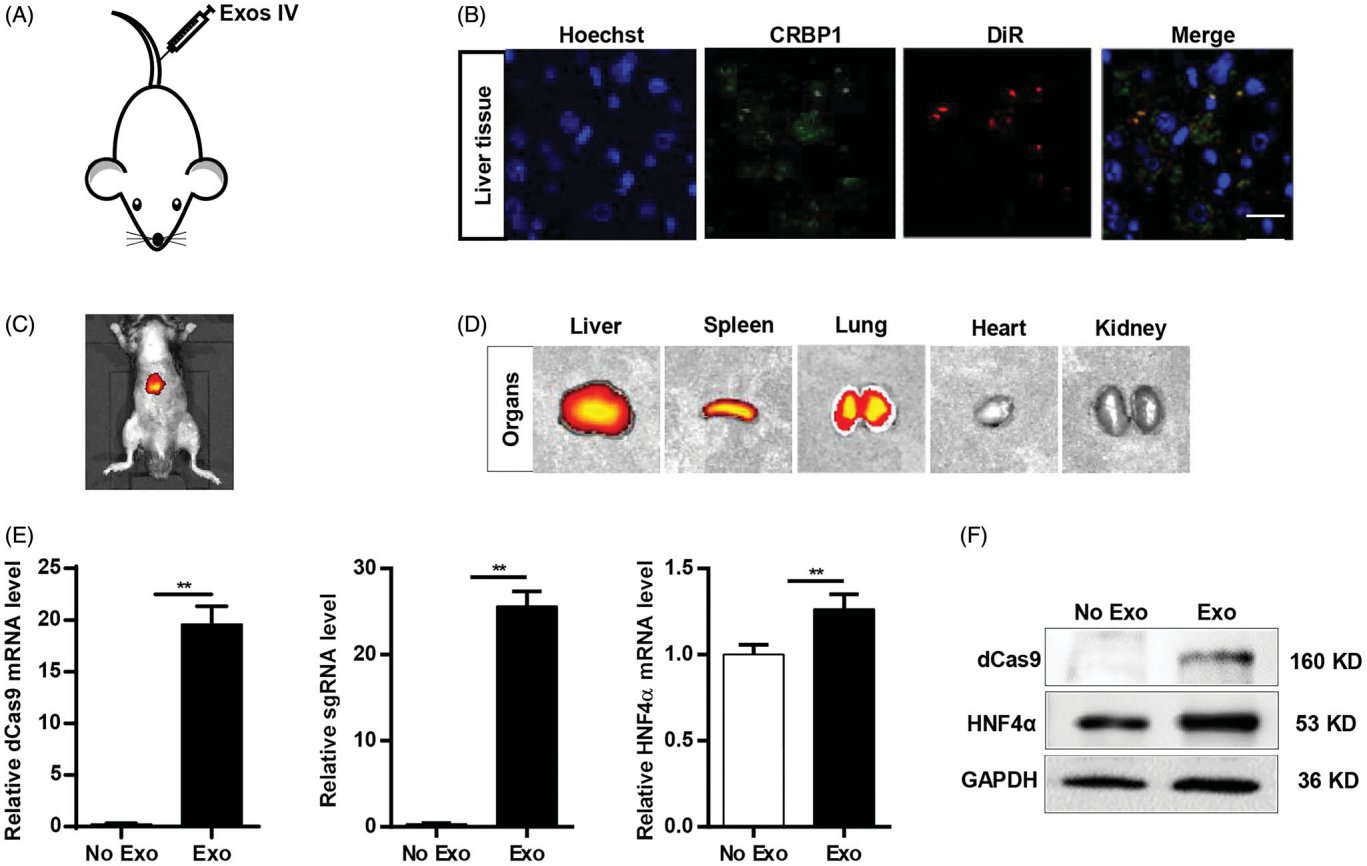
AML12-Exos effectively deliver CRISPR/dCas9-VP64 system to liver in vivo. (A) Schematic representation of the exosomes injection procedure via tail vein; (B) immunofluorescence microscope image of the colocation of DiI-labeled exosomes and CRBP1 in HSCs of liver tissues (scale bar = 20 μm). Nuclei were counterstained with Hoechst; (C, D) liver and lung were the dominated organs of the DiR-labeled exosomes as revealed by bioluminescence imaging; (E) expression of dCas9 mRNA, sgRNA, HNF4α mRNA in the liver tissues treated as indicated was analyzed by qPCR. Data are expressed as mean ± SEM of three different experiments. **p < .01; (F) western blot analysis of the expression of dCas9 and HNF4α in the liver tissues treated as indicated. GAPDH served as the loading control.

To further verify whether AML12-Exos could deliver the CRISPR/dCas9-VP64 system in vivo, DiI/DiR labeled exosomes were injected into mouse via tail vein (Figure 6(A)). Consistently, immunofluorescence microscope analysis found that DiI-labeled exosomes and CRBP1 colocated in the cytoplasm of HSCs in the liver tissues (Figure 6(B)). In vivo imaging revealed that the liver and lung were the dominated organs of the DiR-labeled exosomes (Figure 6(C,D)), suggesting that the exosome delivery to the liver after in vivo injections is specific. qPCR and western blot analysis revealed that dCas9 mRNA, sgRNA, HNF4α mRNA level and protein HNF4α and dCas9 significantly increased in the liver tissues compared with the no exosomes control (Figure 6(E,F)). All the data suggest that the CRISPR/dCas9-VP64 system could be efficiently delivered in liver, and thus might be translational in vivo.

### 3.2. Discussion

Liver fibrosis is a serious threat to human health (Schattenberg, 2019). Currently, although the mechanism of the occurrence and development of hepatic fibrosis has been well understood, there are no clinical drugs that can effectively treat liver fibrosis (Lee et al., 2015). Due to the lack of drug delivery targeting, the therapeutic effect of liver fibrosis is limited and often accompanied by side effects. In order to improve the therapeutic effect of liver fibrosis, targeting delivery using nanocarriers has been widely studied. HSCs are effector cells for the occurrence and development of liver fibrosis (Tsuchida & Friedman, 2017). In recent years, the research on nanometer delivery vectors for targeted hepatic stellate cells to treat hepatic fibrosis has provided a new idea for target gene therapy of liver fibrosis.

Gene therapy is to introduce exogenous normal genes into human target cells in a certain way to correct or compensate for diseases caused by gene defects and abnormalities, so as to achieve the goal of treatment (Yla-Herttuala, 2017). Gene editing technology is a precise site-specific modification technology for genome, which can knock out, add and replace specific DNA fragments, so as to carry out precise gene editing at the genome level. This technology has become a powerful tool for gene function, genomics, gene therapy and other aspects. CRISPR/dCas9 gene editing technology is based on the bacteria or the archaea CRISPR mediated the acquired immune system derived a new type of gene editing techniques, identified by a complementary RNA by base pairing of DNA, guiding Cas9 nuclease cutting identification of double-stranded DNA, induce homologous recombination or non-homologous end link, achieve the purpose of DNA for editing (Lin et al., 2018). Compared with the traditional gene editing system, this system has the advantages of more efficient, simple operation and low cytotoxicity, and has become the most widely used genome editing tool. Currently, CRISPR/dCas9 gene editing technology has been applied in many aspects of disease research, including research on gene function, gene resistance, and building animal models (Chen et al., 2019). The CRISPR/dCas9 system can achieve efficient gene knockout, gene knockin, gene upregulation and gene downregulation, etc. However, there are still some other challenges for the application of CRISPR/dCas9 system in clinical treatment, such as the off-target effect and low delivery efficiency (Li et al., 2019).

Exosomes are nanometer bilayer lipid membrane vesicles released by a variety of cells in the body, which have been found in a variety of body fluids, including blood, urine, bronchoalveolar lavage fluid, and even in saliva and milk (Wiklander et al., 2019). Exosomes, which carry a variety of bioactive substances such as proteins, nucleic acids and lipids, are important carriers of intercellular communication in microenvironment and closely related to the occurrence, development, invasion and metastasis of various diseases. Exosome as an important carrier of signal communication and substance transfer between cells, plays an important role in early diagnosis, treatment and prognosis detection of diseases. However, exosome as a target delivery vector still needs improvement, such as exosome isolation, purification, preservation, loading efficiency and targeting specificity (Leidal et al., 2020). It is believed that with the deepening of research, exosomes may be able to make new breakthroughs in the occurrence, development and treatment of hepatic fibrosis. In the near future, exosomes are expected to be used as natural carriers of targeted therapy through nano-engineering methods, loaded with therapeutic agents and targeted peptides, so as to directly and specifically realize targeted therapy for liver fibrosis. Here, we explored the possibility whether exosomes could deliver the CRISPR/dCas9-VP64 system to HSCs. It came out that the AML12-Exos did encapsulate and deliver the CRISPR/dCas9-VP64 system successfully.

## 4. Conclusions

In conclusion, the findings indicate the potential use of AML12 cell-derived exosomes for encapsulating and delivering the CRISPR/dCas9-VP64 system. However, further efforts will be made for improving encapsulating efficiency and targeting specificity of this delivery system. What is more, there still exist some challenges for in vivo application.

## Supplementary Material

Supplemental Material
