# Supplementary material for: Hepatic stellate cell reprogramming via exosome-mediated CRISPR/dCas9-VP64 delivery
Source: Drug Deliv. 2020 Dec 18;28(1):10–8. doi: 10.1080/10717544.2020.1850917 (PMC7751418; doi:10.1080/10717544.2020.1850917)
Supplement: Supplemental Material [file IDRD_A_1850917_SM8486.docx]

**Supplementary Information**

**Hepatic stellate cell reprogramming via exosome-mediated CRISPR/dCas9-VP64 delivery**

**Nianan Luo^a,b^,** **Jiangbin Li^a^, Yafeng Chen****^a^,** **Ya****n Xu^a^, Yu Wei^c^, Jianguo Lu^a^ and** **Rui** **Dong^a^**

^a^Department of General Surgery, Tangdu Hospital, Fourth Military Medical University, Xi’an, China

^b^Department of General Surgery, 943 Hospital of PLA, Wuwei, China;

^c^Department of Breast Surgery, Enshi Central Hospital, Enshi, Hubei, China


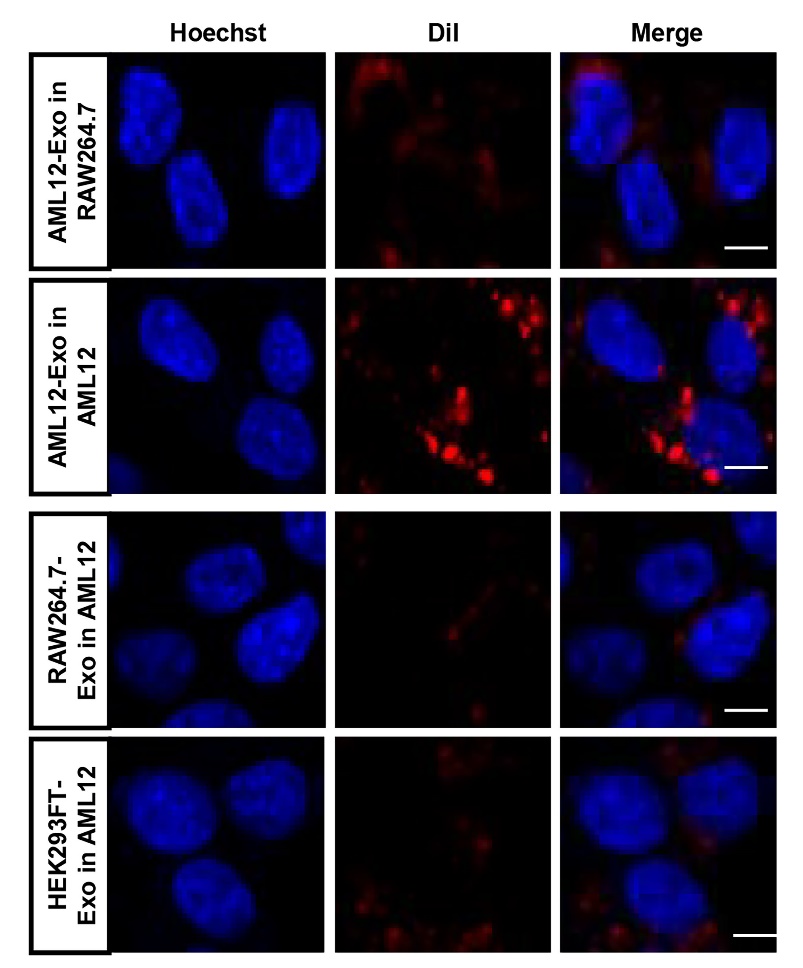


**Figure S1.** Immunofluorescence microscope analysis of the endocytosis efficiency of exosomes derived from different cells. Nuclei were counterstained with Hoechst. Scale bar=5 μm.

| Primer | Sequence |
| --- | --- |
| qPCR primers | |
| *Hnf4α F* | 5’-CACGCGGAGGTCAAGCTAC-3’ |
| *Hnf4α R* | 5’-CCCAGAGATGGGAGAGGTGAT-3’ |
| *dCas9 F* | 5’-GTGGACGCTATCGTGCCTCAG-3’ |
| *dCas9 R* | 5’-GCCGCCAGTAGTTCTTCATCTT-3’ |
| *Hnf4α gRNA F* | 5’-GAGGAGTTTTAGAGCTAGGCCA-3’ |
| *Hnf4α gRNA R* | 5’-TGGCCAAGTTGATAACGGAC-3’ |
| *Gapdh F* | 5’-AGGTCGGTGTGAACGGATTTG-3’ |
| *Gapdh R* | 5’-TGTAGACCATGTAGTTGAGGTCA-3’ |
| Primers for plasmid construction | |
| *Hnf4α gRNA clone F* | 5’-CACCGGACAGTTTGAAAGAGAGGAG-3’ |
| *Hnf4α gRNA clone R* | 5’-AAACCTCCTCTCTTTCAAACTGTCC-3’ |
| *NC gRNA clone F* | 5’-CACCGCCACTTGGCCTAAGGGTT-3’ |
| *NC gRNA clone R* | 5’-AAACCTCTCTCCACTAGCACTATGC-3’ |

**Table S1.** Sequences of the primers used in the study.
